# First co-expression of a lipase and its specific foldase obtained by metagenomics

**DOI:** 10.1186/s12934-014-0171-7

**Published:** 2014-12-16

**Authors:** Viviane Paula Martini, Arnaldo Glogauer, Marcelo Müller-Santos, Jorge Iulek, Emanuel Maltempi de Souza, David Alexander Mitchell, Fabio Oliveira Pedrosa, Nadia Krieger

**Affiliations:** Departamento de Química, Universidade Federal do Paraná, Cx. P. 19081 Centro Politécnico, Curitiba, 81531-980 Paraná Brazil; Instituto Federal do Paraná - Campus Irati, Rua Pedro Koppe, 100, Irati, 84500-000 Paraná Brazil; Departamento de Bioquímica e Biologia Molecular, Universidade Federal do Paraná, Cx. P. 19046, Centro Politécnico, Curitiba, 81531-980 Paraná Brazil; Agência Tecpar de Inovação, Instituto de Tecnologia do Paraná – Tecpar, Curitiba, 81350-010 Paraná Brazil; Departamento de Química, Universidade Estadual de Ponta Grossa, Av. Carlos Cavalcanti, 4748, Ponta Grossa, 84070-900 Paraná Brazil

**Keywords:** Lipases, Metagenomics, Biocatalysis, Lipase-foldase co-expression

## Abstract

**Background:**

Metagenomics is a useful tool in the search for new lipases that might have characteristics that make them suitable for application in biocatalysis. This paper reports the cloning, co-expression, purification and characterization of a new lipase, denominated LipG9, and its specific foldase, LifG9, from a metagenomic library derived from a fat-contaminated soil.

**Results:**

Within the metagenomic library, the gene *lipg9* was cloned jointly with the gene of the foldase, *lifg9.* LipG9 and LifG9 have 96% and 84% identity, respectively, with the corresponding proteins of *Aeromonas veronii* B565. LipG9 and LifG9 were co-expressed, both in N-truncated form, in *Escherichia coli* BL21(DE3), using the vectors pET28a(+) and pT7-7, respectively, and then purified by affinity chromatography using a Ni^2+^ column (HiTrap Chelating HP). The purified enzyme eluted from the column complexed with its foldase. The molecular masses of the N-truncated proteins were 32 kDa for LipG9, including the N-terminal His-tag with 6 residues, and 23 kDa for LifG9, which did not have a His-tag. The biochemical and kinetic characteristics of the purified lipase-foldase preparation were investigated. This preparation was active and stable over a wide range of pH values (6.5-9.5) and temperatures (10-40°C), with the highest specific activity, of 1500 U mg^−1^, being obtained at pH 7.5 at 30°C. It also had high specific activities against tributyrin, tricaprylin and triolein, with values of 1852, 1566 and 817 U mg^−1^, respectively. A phylogenetic analysis placed LipG9 in the lipase subfamily I.1. A comparison of the sequence of LipG9 with those of other bacterial lipases in the *Protein Data Bank* showed that LipG9 contains not only the classic catalytic triad (Ser^103^, Asp^250^, His^272^), with the catalytic Ser occurring within a conserved pentapeptide, Gly-His-Ser-His-Gly, but also a conserved disulfide bridge and a conserved calcium binding site. The homology-modeled structure presents a canonical α/β hydrolase folding type I.

**Conclusions:**

This paper is the first to report the successful co-expression of a lipase and its associated foldase from a metagenomic library. The high activity and stability of Lip-LifG9 suggest that it has a good potential for use in biocatalysis.

**Electronic supplementary material:**

The online version of this article (doi:10.1186/s12934-014-0171-7) contains supplementary material, which is available to authorized users.

## Background

Lipases can be used as biocatalysts in the synthesis of value-added esters for use in the cosmetic [1],[2] and aroma industries [3]-[5], in the enzymatic synthesis of biodiesel [6]-[12], in the modification of fats and oils [13] and to carry out stereospecific reactions in the synthesis of pharmaceuticals and other high-value compounds [14]-[16]. In many of these applications water is not the solvent, with the reaction being carried out in hydrophobic solvents, although hydrophilic solvents such as methanol or ethanol may be present as substrates [17],[18]. These solvents can negatively affect activity and cause high rates of denaturation. This has prompted researchers to search for new esterases and lipases that have high activity and stability in organic solvents. To date, this has mostly been done through traditional techniques of isolation and cultivation of microorganisms, but there are several recent reports involving the metagenomic approach, in which genes have been isolated directly from environments such as bovine rumen microflora [19], mud and sediment-rich water in thermal environments [20],[21], oil-contaminated soils [14],[22]-[25], soils and compost [26],[27], deep-sea sediments [28]-[31], arctic sediments [32],[33], leachates [34] and soils from other ecosystems [22],[35]-[41].

The metagenomic approach involves heterologous expression and a key issue in producing lipases through heterologous expression is to obtain an active enzyme. In some cases, the correct folding of the lipase requires the presence of a specific foldase. This is the case, for example, with the lipases of the gram-negative bacteria *Pseudomonas* and *Burkholderia*, which belong to lipase subfamilies I.1 and I.2 [42],[43].

Studies of co-expression of lipases and their foldases are still scarce. There are two possible strategies for obtaining foldase-assisted folding. Firstly, *in vitro* folding can be used, in which the foldase is produced separately and added to a crude or purified extract. Some success has been obtained using this approach [44]-[47]. Secondly, *in vivo* folding can be used, in which traditional molecular biology techniques are used to co-express the foldase with the lipase [46]. However, in the case of the metagenomic approach, the co-expression is more complicated, since it requires the cloning of both the lipase and foldase on the same DNA fragment. This has not previously been reported in the literature.

In our work, we describe, for the first time, the co-expression, in *Escherichia coli*, of a new metagenomic lipase, LipG9, with its specific foldase, its purification and biochemical and kinetic characterization.

## Results

### Library, activity screening, sequencing and preliminary sequence analysis

In previous work, Glogauer et al. [24] constructed a metagenomic library, consisting of 500 000 clones, from a sample of fat-contaminated soil from an anaerobic lagoon of the wastewater treatment plant of a meat packing and dairy industry located in the state of Paraná, Brazil. Of these clones, 127 showed activity against tricaprylin and 32 showed activity against triolein. In the present work, one of the clones with activity against triolein, pCC2FOS-*lipg9-lifg9*, was selected for expression and characterization.

Sequencing of pCC2FOS-*lipg9-lifg9* identified a 2708 bp contig, which aligned to *Aeromonas veronii* lactonizing lipase with 47% coverage and 99% identity. Within the contig, a 921 bp lipase gene (*lipg9*) and a 723 bp foldase gene (*lifg9*), with 96% and 84% identity, respectively, with the corresponding genes from *Aeromonas veronii* B565 [GenBank:10486164, GenBank:10486163], were identified. The TMHMM server [48] identified a putative transmembrane α-helix for the foldase, while SignalP [49] identified both a signal peptide and a putative cleavage site for the lipase. These results suggest that LipG9 is secreted using the *Sec* mechanism, as reported for other bacterial lipases, such as those from *Pseudomonas* spp. [42],[50],[51], *Burkholderia* spp. [42],[52],[53] and *Aeromonas* spp. [54].

### Cloning strategy, co-expression, purification and mass spectrometry analysis

On the basis of the sequence results, primers were designed for the genes *lipG9* and *lifG9*, both for the sequences of the entire genes and for sequences in which, respectively, the first 75 nucleotides (corresponding to 25 amino acids) and 99 nucleotides (corresponding to 33 amino acids) were deleted. These N-truncated sequences simulated the predicted *in vivo Sec* mechanism of secretion for LipG9. In the expression studies undertaken with these constructs, no lipolytic activity was detected in the culture medium when LipG9 was expressed alone in *E. coli* BL21(DE3), nor when LipG9 was cloned and co-expressed with its foldase, with LipG9 having a His-tag on the C-terminal, this being true for both the entire and the N-truncated constructs (Table 1). On the other hand, when LipG9 and LifG9 were co-expressed, with LipG9 having a His-tag on its N-terminal, lipolytic activity was detected in the medium for both the entire and the N-truncated constructs (Table 1), which gave specific activities against tricaprylin of up to 12 U mg^−1^. The construct in which the N-terminals of both the lipase and its foldase were deleted was selected for the overexpression and purification of the complex Lip-LifG9.

**Table 1:** **Co-expression assays and lipase activity of the constructs**

Construct	Vectors	Lipase (LipG9)	Location of His-Tag in LipG9	Foldase (LifG9)	Activity (U mg ^−1^) ^*^
# 1	pET29b(+)	Entire	C-terminal	-	-
# 2	pET29b(+)	N-truncated	C-terminal	-	-
# 3	pET28a(+)	Entire	N-terminal	-	-
# 4	pET28a(+)	N-truncated	N-terminal	-	-
# 5	pT7-7	-	-	Entire	-
# 6	pT7-7	-	-	N-truncated	-
# 1-5	pET29b(+) and pT7-7	Entire	C-terminal	Entire	-
# 1-6	pET29b(+) and pT7-7	Entire	C-terminal	N-truncated	-
# 2-5	pET29b(+) and pT7-7	N-truncated	C-terminal	Entire	-
# 2-6	pET29b(+) and pT7-7	N-truncated	C-terminal	N-truncated	-
# 3-5	pET28a(+) and pT7-7	Entire	N-terminal	Entire	12 ± 1
# 3-6	pET28a(+) and pT7-7	Entire	N-terminal	N-truncated	2 ± 0
# 4-5	pET28a(+) and pT7-7	N-truncated	N-terminal	Entire	1 ± 0
# 4-6	pET28a(+) and pT7-7	N-truncated	N-terminal	N-truncated	11 ± 1

*The activity was determined by the titrimetric method with tricaprylin as the substrate, using a pHStat at pH 7.5 and 30°C. Results are expressed as the average of triplicates assays ± the standard error of the mean.

**Figure 1:**
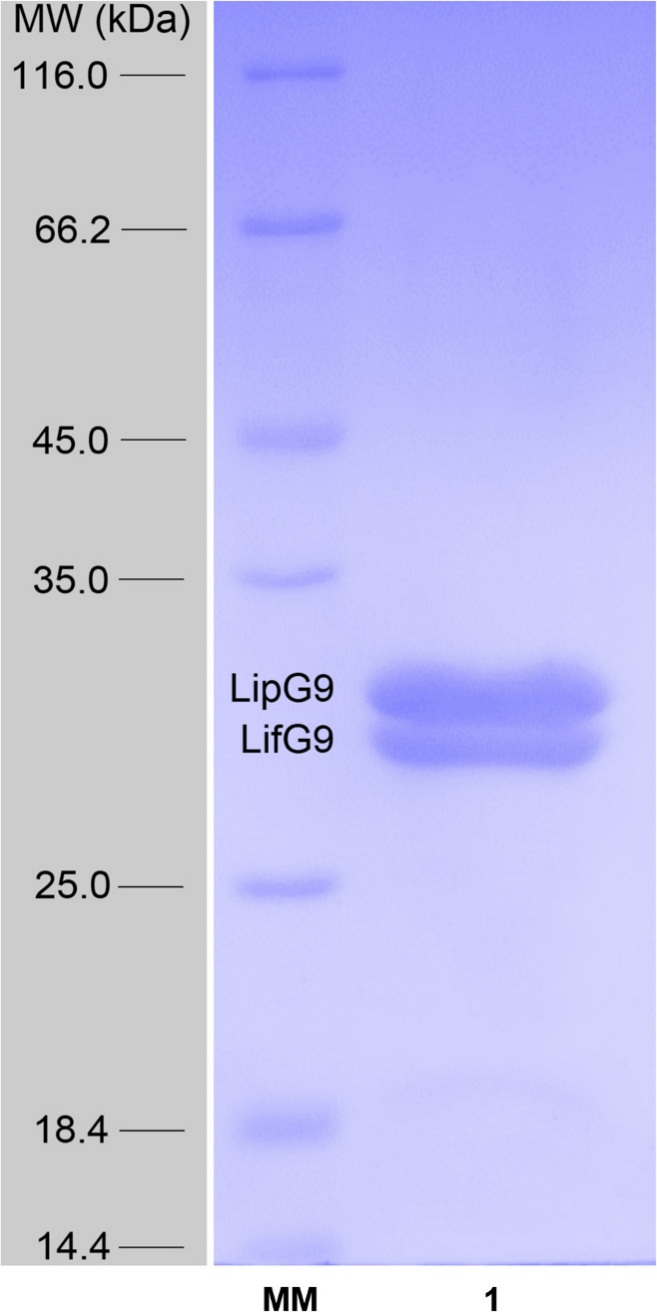
**Purification of active LipG9.** SDS-PAGE of the lipase (LipG9) and foldase (LifG9) fractions as eluted from the affinity chromatography column (Lane 1). Lane MM, protein molecular weight markers.

During purification, the lipase and foldase were co-eluted from the affinity column when imidazol was in the concentration range of 0.208 mol L^−1^ to 0.280 mol L^−1^. Since only the lipase had a His-tag, the foldase must have been complexed to the lipase that bound to the support. The two bands on the SDS-PAGE gel (lane 1, Figure 1) correspond to the lipase and the foldase, for which ProtParam [48] had predicted theoretical molecular masses of 32 kDa and 24 kDa, respectively. The migration of the lipase was consistent with its theoretical molecular mass. However, the migration of the foldase was aberrant, giving a higher than expected apparent molecular mass, 31 kDa. According to the densitometry analysis, the bands in the SDS-PAGE were 95% pure. As the bands gave approximately the same density, it can be deduced that the complex is eluted from the affinity column in a 1:1 proportion of LipG9 and LifG9.

The sequences obtained through mass spectrometry (MALDI-TOF) confirmed that LipG9 and LifG9 were N-truncated. The fragment masses in the mass spectra were compared to theoretical masses predicted by *in silico* peptide cleavage using ProteinProspector [55], giving sequence coverages of 25% for the lipase and 39% for the foldase (data not shown). The sequence coverages and the fragment masses of both LipG9 and LifG9 confirmed the identity of the purified enzyme complex.

### Protein sequence and protein 3D structure model analyses

**Figure 2:**
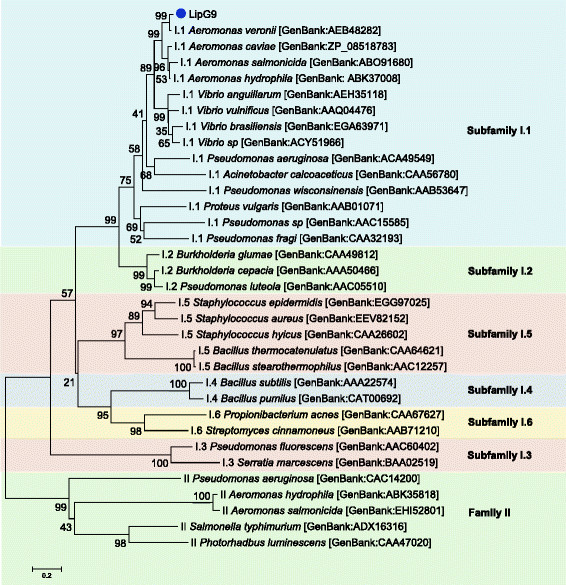
**Phylogenetic analysis of LipG9 and closely related proteins.** The enzymes most similar to LipG9 are from *Aeromonas* spp. and *Vibrio* spp. LipG9 is a member of subfamily I.1 [43]. Except for LipG9, the protein sequences were retrieved from GenBank (NCBI). The phylogenetic tree was generated using MEGA 6 [56]. The scale represents the number of amino acid substitutions per site.

The phylogenetic analysis of LipG9 indicates that it belongs to lipase subfamily I.1 (Figure 2), the same subfamily as that of the lipases from *Pseudomonas aeruginosa* [GenBank:ACA49549.1], *Aeromonas hydrophila* [GenBank:ABK37008.1] and *Aeromonas veronii* [GenBank:AEB48282.1]. Of these three lipases, it has highest identity, 96%, with the last one.

**Figure 3:**
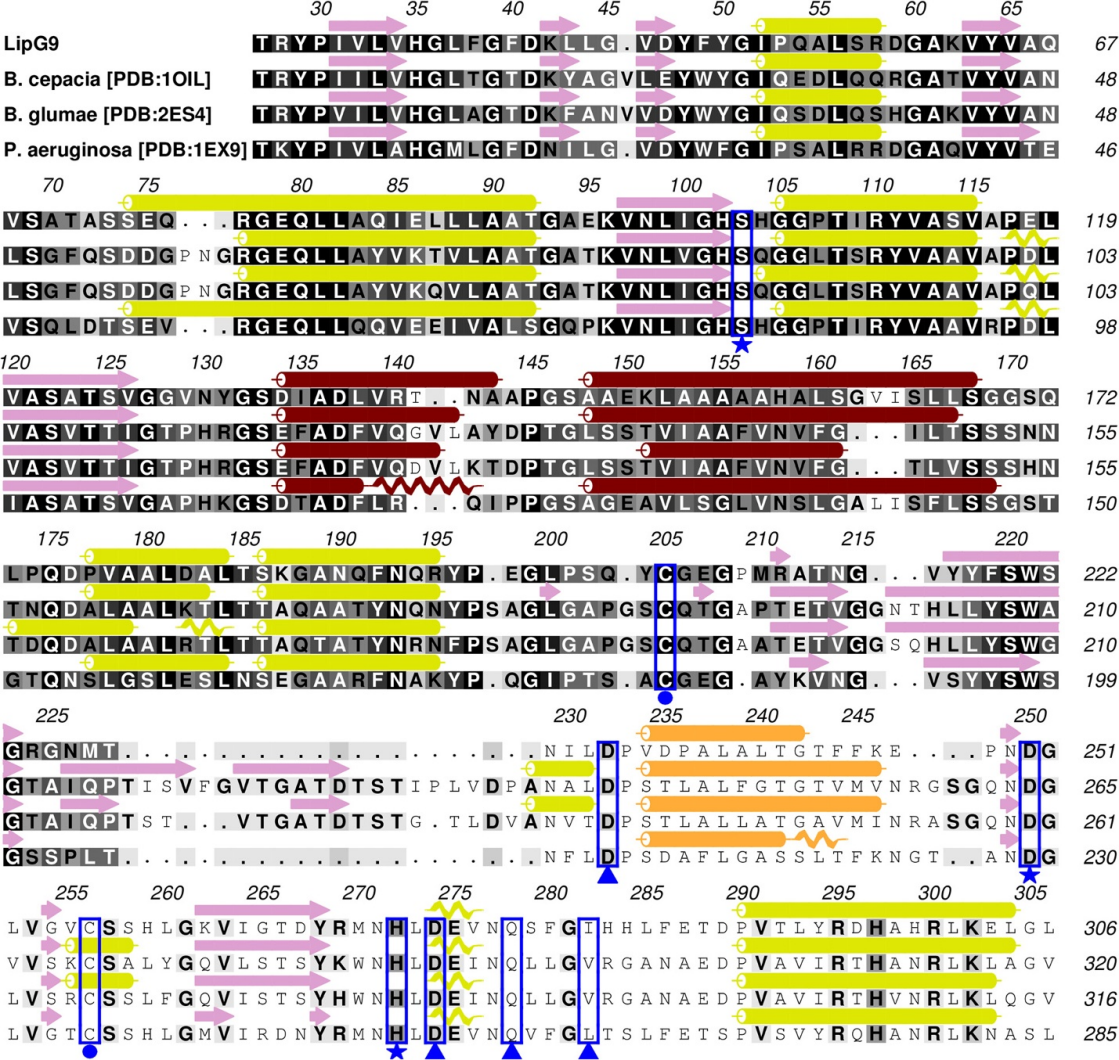
**Secondary structure estimation and sequence alignment to available structures.** Stars indicate the catalytic triad, triangles indicate the residues in the putative binding site for Ca^2+^ and circles indicate disulfide bonds. The background is colored according to the convention ALSCRIPT Calcons [58]. Representations of secondary structures are given above the sequences: i) arrows for β-strands (in mauve) and ii) cylinders for α-helices (brown cylinders indicate movable helices in the catalytic site, orange cylinders indicate fixed helices in the catalytic site and yellow-green cylinders indicate the remaining helices).

The LipG9 sequence was aligned with the sequences of lipases that are available in the PDB (Protein Data Bank) [57]. LipG9 has sequence identities of 72% with the lipase from *Pseudomonas aeruginosa* (PDB:1EX9), 54% with the lipase from *Burkholderia cepacia* (PDB:1OIL) and 51% with the lipase from *Burkholderia glumae* (PDB:2ES4). Figure 3 shows the alignment and the estimated secondary structures. These structures were used as templates for the structural homology modeling.

The N-termini of LipG9 and of the structures from the PDB were not aligned due to the fact that the PDB sequences do not show ordered crystal structures for the N-terminal regions. The alignment suggests that LipG9 possesses the conserved sequential pentapeptide Gly-Xaa-Ser-Xaa-Gly, which is typical of lipases of subfamily I.1 [43], with both Xaa corresponding to His residues, centered on Ser^103^ (Figure 3). The other two residues of the catalytic triad were identified as Asp^250^ and His^272^. These two residues are in the C-terminal region and they are also part of the α/β hydrolase fold that is typical of lipases.

The alignment of LipG9 with PDB structures also suggests that Asp^232^, Asp^274^, Gln^278^ and Ile^282^ form a calcium binding pocket, which is noteworthy, since lipases that have a bound calcium usually have higher activities and stability than those that do not [59]. LipG9 also contains two cysteine residues (Cys^205^ and Cys^256^) that are predicted to form a disulfide bridge, analogous to that which occurs in the lipases of *A. hydrophila*, *A. salmonicida* and other lipases used as templates in the alignment (Figure 3)*.* The presence of the disulfide bridge suggests that LipG9 may be extracellular in the original microorganism [60].

The final LipG9 structural homology models were built with 286 amino acids and the calcium ion. The consistency of the proposed models was extensively checked. The model with the lowest normalized DOPE score (−1.064) and a GA341 score of 1.000 (which indicated a correct fold) was chosen for later analyses. This DOPE score is close to those of the templates (−1.816, −1.507 and −1.193 for 2ES4, 1EX9 and 1OIL, respectively). The program PROCHECK identified three Ramachandran outliers; two of the outlying residues occur in loops and have conformations close to the limits of allowed regions on the Ramachandran plot. The other outlier is Ser^103^, which is predicted to have the same conformation as the corresponding Ser residues in the templates. The domain analysis carried out using the Pfam database showed that LipG9 probably has a canonical α/β hydrolase folding type I, between residues Gly^23^ and Lys^246^.

**Figure 4:**
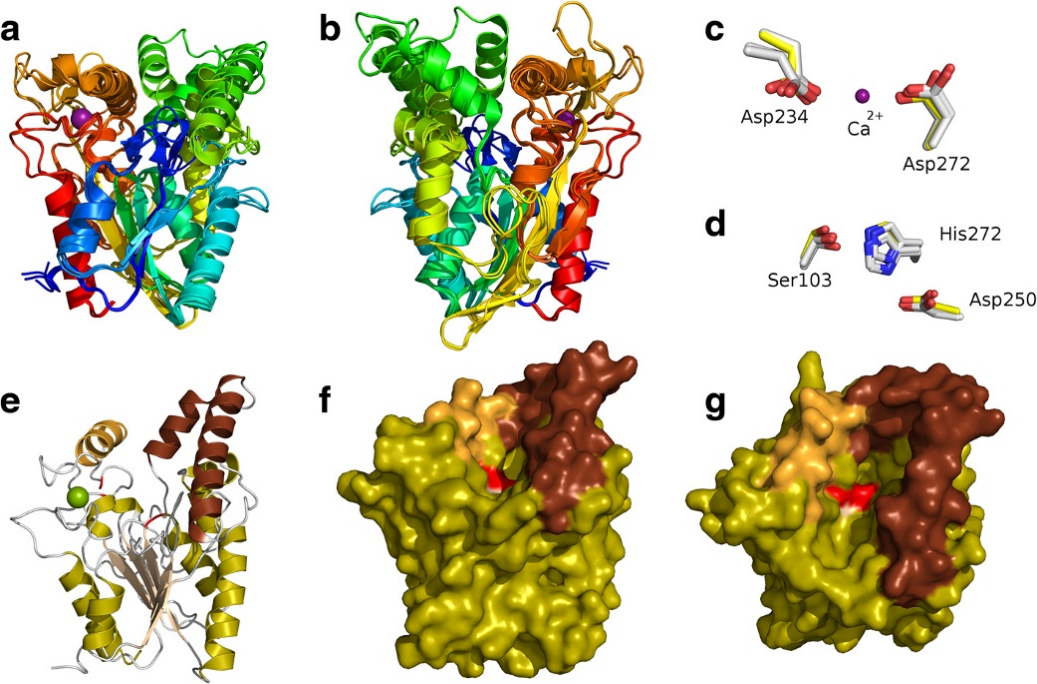
**Homology model of LipG9. a)** Homology-modeled LipG9 structure superimposed on the template structures. **b)** The same as Figure 4a, but rotated 180°. **c)** The calcium ion and its coordinating aspartate residues. **d)** The catalytic triad. **e)** The modeled structure, in the same orientation as in Figure 4a, but with its secondary structure highlighted according to Figure 3. **f)** A space filling model of Figure 4e. **g)** The same as Figure 4f, but rotated 45°.

To explore the structural characteristics of LipG9 further, its model was superimposed on the structures of the three lipases used as templates. As shown in Figure 4, LipG9 and these three lipases have very similar conformations. This structural modeling confirmed key features that were predicted above for LipG9 by other methods, including the canonical α/β hydrolase fold (Figure 4, parts a and b), the calcium binding pocket (Figure 4, parts c and e) and the disulfide bridge. With respect to the calcium binding pocket, the model confirms that the carboxylate groups of Asp^232^ and Asp^274^ are in the correct positions to coordinate the calcium ion adequately (Figure 4c). With respect to the disulfide bridge, the model confirms that Cys^205^ and Cys^256^ are adequately positioned to form this bridge. Additionally, the modeled LipG9 structure shows the lid subdomain, which is composed of four α-helices, in an open conformation that is similar to the open-lid conformations of the lipase structures of *B. cepacia* (1OIL) and *P. aeruginosa* (1EX9) (Figure 4, parts f and g).

### Hydrolytic activity of Lip-LifG9 against triacylglycerols

The activity of the purified preparation of LipG9 and LifG9 was studied. This preparation will be referred to from this point as Lip-LifG9. Although the lipase and foldase were eluted from the affinity column in complexed form and may have remained as a complex throughout this part of the work, it should be noted that we did not undertake any studies to confirm that the two proteins in fact remained bound in a complex.

**Figure 5:**
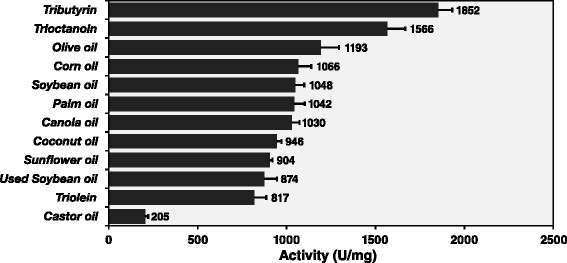
**Enzymatic activity of Lip-LifG9 obtained using the titrimetric method with triacylglycerols.** Assays were carried out using a pHStat, at 30°C, with an emulsion containing 67 mmol L^−1^ of the substrates. The pH was maintained at 7.5. The assays were done in triplicate. The error bars represent the standard error of the mean.

The highest specific activity of Lip-LifG9 was against tributyrin (1852 U mg^−1^). The specific activity decreased with increasing length of the carbon chain, from 1566 U mg^−1^ for tricaprylin to 817 U mg^−1^ for triolein (Figure 5). Specific activities were between 870 and 1190 U mg^−1^ for all commercial oils tested, except for castor oil, with which Lip-LifG9 had a specific activity of only 205 U mg^−1^, probably because of the unusual hydroxyl group on the twelfth carbon of ricinoleic acid.

### Effect of temperature on Lip-LifG9 activity and stability

**Figure 6:**
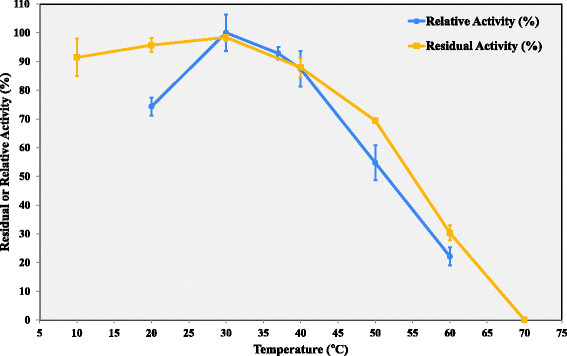
**Effect of temperature on Lip-LifG9 activity and stability.** In both studies, activity was determined by the titrimetric method with tricaprylin as the substrate, while in all incubations and assays the pH was 7.5. For the activity study, relative activities are plotted, with 100% corresponding to the activity at 30°C. For the stability study, Lip-LifG9 was incubated for 1.5 h before determination of the residual activity and 100% corresponds to the activity after incubation at 30°C. The assays were done in triplicate. The error bars represent the standard error of the mean.

The highest specific activity of Lip-LifG9 for the hydrolysis of tricaprylin was obtained at 30°C (1566 ± 101 U mg^−1^). At 50°C, its activity was only half this value and at 60°C it was only 30% of this value (Figure 6). In stability studies involving previous incubation of Lip-LifG9 for 1.5 h at 10-40°C without substrate, residual activities of around 90% or better were obtained. At the higher temperatures of 50°C and 60°C, the residual activities were around 70% and 30%, respectively (Figure 6).

### Effect of pH on Lip-LifG9 activity and stability

**Figure 7:**
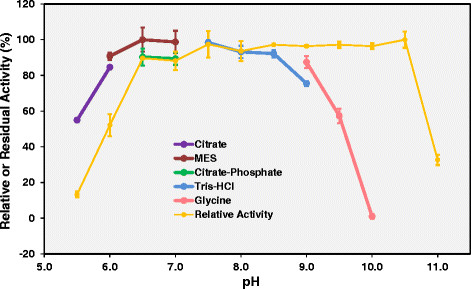
**Effect of pH on Lip-LifG9 activity and stability.** In both studies, activity was determined by the titrimetric method with tricaprylin as the substrate, while in all incubations and assays the temperature was 30°C. For the activity study, relative activities are plotted (yellow line), with 100% corresponding to the activity at pH 7.5. For the stability study, Lip-LifG9 was incubated for 1.5 h in various buffers (all at 50 mmol L^−1^), as indicated by the other colored lines; residual activities were then determined at pH 7.5, with 100% corresponding to the activity after 1.5 h incubation in Tris–HCl buffer at pH 7.5. The assays were done in triplicate. The error bars represent the standard error of the mean.

Lip-LifG9 had high specific activities (around 1500 U mg^−1^) from pH 6.5 to 10.5 (Figure 7). In the stability study, residual activities were highest after incubation at pH values from 5.5 to 9.5. The composition of the buffer affected stability: Lip-LifG9 was more stable at pH 5.5 when incubated in 2-(N-morpholino)ethanesulfonic (MES) buffer (100% residual activity) than in citrate-phosphate buffer (50% residual activity).

### Stability of Lip-LifG9 in organic solvents

**Figure 8:**
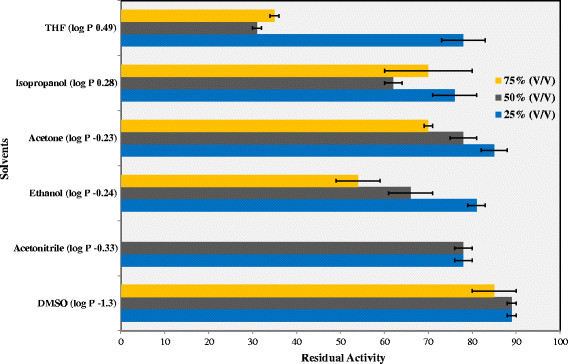
**Stability of Lip-LifG9 in hydrophilic organic solvents.** After incubation of Lip-LifG9 for 1.5 h in the solvents at 25, 50 and 75% concentration, residual activities were determined by the titrimetric method with tricaprylin as the substrate, using a pHStat at pH 7.5 and 30°C. In this case, 100% corresponds to the residual activity when Lip-LifG9 was incubated in 50 mmol L^−1^ Tris–HCl buffer, pH 7.5. The assays were done in triplicate. The error bars represent the standard error of the mean.

Lip-LifG9 was incubated, at 30°C for 1.5 h, in different concentrations of various organic solvents. With the exception of DMSO, the residual activity of Lip-LifG9 after incubation decreased with increasing solvent concentration (Figure 8). Even so, in the case of acetone, isopropanol and ethanol, Lip-LifG9 presented residual activities of over 50% after incubation in concentrations from 50 to 75%. Such stability is desirable for biocatalytic applications, since these solvents are used in many organic syntheses that require hydrophilic solvents. With DMSO, Lip-LifG9 had over 85% residual activity for all studied concentrations.

## Discussion

### The importance of the co-expression strategy used for LipG9-LifG9

Researchers have faced difficulties with the expression of active enzymes in heterologous systems when prospecting for new enzymes, with the formation of inclusion bodies being one of the most frequently reported obstacles [46],[61]-[65]. Obtaining active enzymes has the potential to be even more problematic when the enzyme requires a foldase for proper folding, such as is commonly the case with lipases [42],[66]. In the current work, we used a simple and effective *in vivo* folding strategy, in which the genes of a lipase and a foldase, obtained through the metagenomic approach, were cloned in different plasmids and inserted into a single heterologous host, *E. coli* BL21(DE3). Through this approach, we obtained much higher specific activities than did Madan and Mishra [67], who used a similar approach to co-express a non-metagenomic lipase and its corresponding foldase, from *P. aeruginosa* B2264, in *E. coli* BL21(DE3)*.* They reported an activity of 225 U mg^−1^, using *p*-nitrophenyl palmitate as the substrate. We produced a lipase with higher specific activities, not only against purified triacylglycerols (1560 U mg^−1^ and 817 U mg^−1^ for tricaprylin and triolein, respectively) and but also against commercial oils, such as canola, corn, olive and palm oils (all above 1000 U mg^−1^).

The co-expression strategy that we used has the advantage of being simpler than *in vitro* strategies. For instance, Pauwels et al. [52],[68] expressed the lipase of *B. glumae* and its foldase separately, in homologous and heterologous hosts, respectively, and then combined them during the protein purification step, in which the Ni^2+^ affinity column was loaded with separate extracts from each protein. Additionally, *in vitro* folding is not always particularly effective. Hobson et al. [47] showed this by expressing the lipase from *P. cepacia* DSM 3959 in *E. coli*, denaturing it with urea and then refolding it by dialysis in the presence of its foldase, which was also expressed separately in *E. coli* [47]. Only 5 to 10% of the initial activity of the enzyme was regained after refolding. However, it is not impossible to obtain high specific activities using *in vitro* folding, as shown by Traub et al. [45]. They prepared *E. coli* cell extracts containing denatured N-truncated lipases from *Pseudomonas* sp. and *Chromobacterium viscosum.* After adding the respective foldases, they obtained specific activities against triolein of 3900 U mg^−1^ for the lipase of *Pseudomonas* sp. and 2800 U mg^−1^ for the lipase of *C. viscosum*.

### The classification and structural characteristics of LipG9

The phylogenetic analysis classified LipG9 into the bacterial lipase subfamily I.1, which follows the *Sec* mechanism of secretion. This classification is confirmed by three features of LipG9. First, it has a calcium binding pocket that is absolutely conserved in lipases from subfamily I.1 [69]. Secondly, it has a disulfide bridge that is highly conserved in this subfamily. Thirdly, it requires a foldase, which is characteristic of lipases from subfamily I.1.

In LipG9-LifG9, the active construct contains the His-tag on the N-truncated terminal of the lipase. This His-tag is therefore far from the two residues of the catalytic triad that are located in the C-terminal region, Asp^250^ and His^272^ (Figures 3 and 4). In fact, the complete lack of activity of the co-expressions in which the His-tag was inserted at the C-terminal (Table 1) shows the importance of this region for LipG9 activity. This is not surprising, since this region has been shown to be important for activity of many of the lipases that have been characterized to date [70],[71].

### The kinetic characteristics of Lip-LifG9

The high specific activity of Lip-LifG9 (i.e. a preparation containing both the lipase and the foldase) against olive oil, 1200 U mg^−1^, is comparable to values obtained with the most-used commercial lipases, for instance, those from *Rhizopus oryzae*, *Rhizomucor miehei* and *Thermomyces lanuginosus* (formerly *Humicola lanuginosa*), which have specific activities against olive oil of 1000, 3300 and 2900 U mg^−1^, respectively [72]. The fact that Lip-LifG9 has a high specific activity against this long chain substrate makes it a true metagenomic lipase [73],[74]. Of the many hydrolytic enzymes isolated from metagenomics with claimed lipase activity [14],[27],[29],[31],[64],[75]-[78], only a few are active against long chain triacylglycerols [19],[24]. Notably, Lip-LifG9 has a higher specific activity against triolein, another long chain triacylglycerol, than do some other true lipases obtained through metagenomics: Lip-LifG9 gave a specific activity of 817 U mg^−1^ with this substrate, while RlipE1 and RlipE2, isolated from a cow rumen metagenomic library [19], gave specific activities of 346 and 232 U mg^−1^, respectively.

Given the origin of the metagenomic library (i.e. fat-contaminated soil from the anaerobic lagoon of a wastewater treatment plant of a meat packing and dairy industry), it is not surprising that the lipase with which LipG9 showed the highest identity was from an *Aeromonas* species. In fact, our results for the activity and stability of LipG9 have similarities with those reported for several lipases from *Aeromonas* species. First, the temperature stability of Lip-LifG9 (Figure 6) is comparable to that of the lipases of *A. caviae* AU04 [79] and *A. sobria* LP004 [80], which kept 100% of their activities up to 40°C during 1.5 h. Secondly, the wide range of pH for activity and stability (6.5 to 9.5) is also characteristic of lipases from *Aeromonas* species (*A. hydrophila*, *A. caviae* AU04) [79],[81]. Thirdly, Lip-LifG9 had good stability in hydrophilic organic solvents such as DMSO, ethanol and isopropanol, with the lipases of *A. caviae* AU04 [79] and *Aeromonas* sp. LPB 4 [82] showing comparable stabilities in isopropanol and ethanol. These characteristics suggest that it is worthwhile to undertake further studies to optimize the activity and stability of LipG9 and to investigate its potential for use in biocatalysis.

## Conclusions

This paper is the first to report the successful co-expression of a lipase and its foldase from a metagenomic library. We used a simple and effective strategy, in which the genes of the lipase and of the foldase were cloned in different plasmids and inserted in a single heterologous host, *E. coli* BL21(DE3), enabling the purification of an active lipase-foldase complex. Phylogenetic analysis suggests that LipG9 belongs to the bacterial lipase subfamily I.1. The most similar sequences are those of the *A. veronii* lipase [GenBank:AEB48282], to which LipG9 has 96% identity, and the *A. veronii* foldase [GenBank:AEB48281], to which LifG9 has 77% identity. The structural model of LipG9 presents both an overall folding and an active site structure that are quite similar to those of the homologue lipases used as templates. The LipG9 model also contains the calcium coordination site and the disulfide bridge that are highly conserved in subfamily I.1 lipases. The high activity of Lip-LifG9 towards various purified triglycerides and commercial oils, its broad range of pH stability and its stability in organic solvents suggest that it has a good potential for use in biocatalysis.

## Methods

### Bacterial strains and plasmids

*Escherichia coli* EPI300™-T1R and pCC2FOS fosmid vector (CopyControl™ Fosmid Library Production Kit, Epicentre Biotechnologies, Madison, USA) were used for constructing the metagenomic library [24]. EZ-Tn5 < KAN-2 > Insertion Kit (Epicentre) and DYEnamic ET Dye Terminator Kit (GE Life Sciences, Uppsala, Sweden) were used for sequencing*.* The NucleoSpin®Extract II PCR purification kit was from Macherey-Nagel GmbH & Co (Düren, Germany). The strains *E. coli* TOP10 (Invitrogen, Carlsbad, CA, USA) and BL21(DE3) (Novagen, Madison, MI, USA) and the vectors pET-28a(+), pET-29b(+) (also from Novagen) and pT7-7 (Addgene, Cambridge, MA, USA) were used as the recombinant protein expression system.

### Chemicals and enzymes

Taq polymerase (MBI Fermentas, Baltimore, MD, USA) was used for DNA amplification. T4 DNA ligase, T4 DNA polymerase, Klenow fragment, T4 polynucleotide kinase, shrimp alkaline phosphatase (SAP), restriction enzymes and the protein molecular mass marker were purchased from Fermentas. The HiTrap Chelating HP column was purchased from GE Healthcare (Uppsala, Sweden). Triolein (65%), tricaprylin (90%) and tributyrin (99%) were purchased from Sigma-Aldrich (St. Louis, MO, USA). The natural oils (olive, corn, canola, palm, sunflower, castor, coconut and soybean) for lipase analysis were commercial products purchased from a local supermarket. All other chemicals used for lipase analysis were of analytical grade.

### Identification and sequencing of the lipase and foldase genes

The clone with the genes *lipg9* and *lifg9*, for the lipase and foldase, respectively, was previously isolated from a metagenomic DNA library built from a fat-contaminated soil [24]. The plasmid DNA was isolated, by the alkaline lysis method [83], for template usage for PCR and for DNA sequencing. For the latter, a collection of derivative plasmids containing randomly inserted EZ-Tn5 < KAN-2 > was obtained by using an *in vitro* transposon insertion reaction with the EZ-Tn5 < KAN-2 > Insertion Kit (Epicentre). These derivative plasmids were then used to generate new clones. Genes from 96 inactive clones were then sequenced with the ABI 377 (Genetic Analyzer, Applied Biosystems/HITACHI, Foster City, CA, USA) and MegaBACE 1000 (GE Healthcare, Uppsala, Sweden) sequencers, from both ends, using DYEnamic ET Dye Terminator Kit (GE Life Sciences). Sequence assembly and editing were performed with the CodonCode Aligner software (CodonCode Corporation, Centerville, MA, USA). The open reading frames (ORFs) were identified with the ORF Finder tool (NCBI) [84].

### Cloning of *lipg9* and *lifg9*genes

Pairs of primers were designed for the lipase and the foldase, both entire and N-truncated, using the TMHMM v.2.0 [85], SignalP 3.0 [49] and ProtScale [48] servers, and obtained from Prodimol Biotechnologies (Belo Horizonte, MG, Brazil). Four lipase and two foldase constructs were prepared. They were first tested when expressed individually (Constructs # 1 to # 6 in Table 1). They were then combined for eight different co-expression experiments (Constructs # 1–5 to # 4–6 in Table 1).

Expression assays were carried out for the individual and associated proteins (i.e. co-expressed in *E. coli*). As shown in Table 1, the His-tag was always on the N-truncated end of active LipG9. The entire and the N-truncated fragments of *lipg9*, with 918 and 843 bp, respectively, and of *lifg9*, with 720 and 621 bp, respectively, were amplified separately by the polymerase chain reaction (PCR), using the fosmidial pCC2FOS-*lip-lifg9* DNA. PCR was performed with Taq polymerase. The resultant PCR product and the expression vectors pT7-7 and pET29b(+) or pET28a(+) were then digested with NdeI and HindIII. The digested PCR product was purified using the NucleoSpin®Extract II PCR purification kit, according to the manufacturer’s instructions. Digested plasmids were purified by phenol chloroform extraction followed by ethanol precipitation [83]. The inserts for entire and N-truncated *lipg9* and *lifg9* were ligated into the vectors. Plasmids were sequenced and then introduced through thermal shock into competent *E. coli* BL21(DE3) cells to express the recombinant LipG9 active lipase.

The *E. coli* TOP10 and BL21(DE3) cells with the genes *lipg9* or *lifg9* were stored at −80°C. Tests for expression of the constructs in *E. coli* BL21(DE3) cells were carried out (Table 1). The cells were collected and then transferred to 75 mL of Luria Bertani (LB) medium, with either 100 mg mL^−1^ kanamycin (*lipg9*) or 50 mg mL^−1^ ampicillin (*lifg9*). After the culture reached an OD_600_ of 0.5, IPTG was added to give a concentration of 0.2 mmol L^−1^ and the culture was continued for another 16 h at 20°C. The supernatant was discarded and the cell pellet was re-suspended in 7 mL of a buffer containing 2.5 mmol L^−1^ Tris–HCl, 4 mmol L^−1^ CaCl_2_, 1% (V/V) Triton X-100 and 150 mmol L^−1^ NaCl. The cells were sonicated in an ice bath ten times for 30 s with an interval of 30 s. The expression of the enzyme was confirmed both by 12% (w/V) SDS-PAGE [86] and by activity assays, done using the titrimetric method with tricaprylin as substrate (90%) in a pH-Stat [87].

### Co-expression and purification of complexed LipG9-LifG9

The lipase and foldase were co-expressed in different combinations (Table 1) within competent *E. coli* BL21(DE3) cells, in which the lipase plasmid was introduced before the foldase plasmid. The assays for activity determination of the co-expression combinations were performed by the titrimetric method with tricaprylin as the substrate, as described below. The best combination was determined as # 4–6 (Table 1) and this combination was further purified.

After sonication, the cell lysate obtained from 2 L of culture medium with the strain *E. coli* BL21(DE3) transformed with combination # 4–6 (Table 1) was loaded onto a HiTrap column equilibrated with 50 mmol L^−1^ Tris–HCl buffer (pH 7.5), containing 5 mmol L^−1^ CaCl_2_, 500 mmol L^−1^ NaCl, 10 mmol L^−1^*β*-mercaptoethanol, 10% (V/V) glycerol, 1% (V/V) Triton X-100 and 10 mmol L^−1^ imidazole. The column was then washed extensively with the same solution. A linear gradient (10–1000 mmol L^−1^) of imidazole in 50 mmol L^−1^ Tris–HCl buffer (pH 7.5) containing 10% (V/V) glycerol at a flow rate of 0.2 mL min^−1^ was then applied and fractions of 1.5 mL were collected. The fractions identified as containing proteins of the expected molecular masses (through SDS-PAGE) were pooled and dialyzed against a buffer consisting of 50 mmol L^−1^ Tris–HCl (pH 7.5), 150 mmol L^−1^ NaCl and glycerol 20% (V/V) for 12 h, with two buffer exchanges.

### Electrophoresis and determination of protein content

Electrophoresis of protein samples was done with 12% (w/V) SDS-PAGE [86]. The gel was stained with Coomassie Brilliant Blue R-250 and destained with methanol/acetic-acid/water (5/1/4, V/V/V). Densitometric analysis of the SDS-PAGE gel was done using the LabWorks Image Acquisition and Analysis software 4.0 (UVP BioImaging Systems, Upland, CA, USA). The protein concentration was determined by the Bradford method [88] using bovine serum albumin (Sigma Chemical Co., St. Louis, MO, USA) as the standard.

### MALDI-TOF/MS analysis

Spots corresponding to the N-truncated lipase and the N-truncated foldase were excised manually from an SDS-PAGE gel for analysis by matrix-assisted laser desorption/ionization (MALDI) time-of-flight (TOF) mass spectrometry (MS). Excised spots were in-gel digested with sequencing grade modified trypsin (Promega, Madison, WI, USA) as described elsewhere [89]. The sample was desalted using a ZipTipC_18_ pipette tip (Millipore Corporation, Billerica, MA, USA) and eluted directly onto the MALDI target plate coated with MALDI matrix (saturated solution of α-cyano-4-hydroxycinnamic acid in 50% (V/V) acetonitrile and 0.1% trifluoroacetic acid). MALDI-TOF data were acquired with an Autoflex II spectrometer (Bruker Daltonics, Bremen, HB, Germany) in the reflector positive ion mode with an acceleration voltage of 20 kV, delay time of 150 ns and acquisition mass range of 800–3200 Da. The mass profiles obtained were compared with the peptide masses predicted by *in silico* digestion of the His-tagged protein sequence using the PeptideCutter and MS-Digest tools [48].

### Lipase sequence analysis and phylogenetic tree construction

To determine the lipase family, the LipG9 sequence was analyzed against the 25 lipase sequences that Arpingy and Jaeger [43] classified into lipase subfamilies I.1 and I.2 This lipase classification scheme is based on conserved sequence motifs and biological properties. Eight other sequences that had a high identity to LipG9, as determined by a BLAST (NCBI) [90], were also aligned. The ProtParam tool was used to calculate the theoretical parameters of the protein [48] and multiple sequence alignment was performed with the ClustalW algorithm [91]. Phylogenetic analysis was carried out with the neighbor-joining method using the MEGA software (version 6). Bootstrapping (10000 replicates) was used to estimate the confidence levels of the phylogenetic reconstructions [56].

A BLAST (NCBI) alignment of the lipase sequence against the PDB [57] identified the sequences with the greatest similarity. The programs DSSP [92], Jpred3 [93] and Aline [94] were used to estimate and to show the predicted secondary structure.

### Structural homology modeling

Homology modeling was carried out using MODELLER software [95]. Suitable experimental structural homologues to use as templates were chosen using the MHOLline algorithm [96] and acquired from the PDB [57]. The templates were superimposed with the Multiprot program [97] and then used to perform sequence alignments with the program T-Coffee [98]. Five hundred models that included the calcium ion were generated and further optimized by means of the variable target function method (VTFM), available within MODELLER. The quality of the models was assessed by the programs PROCHECK [99], WHATCHECK [100] and MODELLER with the embedded normalized DOPE [101] and GA3415 scores [102].

### Titrimetric determinations of lipase activity using triacylglycerols

During the purification procedure described above, LipG9 and LifG9 were co-eluted from the affinity column. This preparation, denominated Lip-LifG9, was used in the investigations of lipase activity.

The substrate emulsions consisted of 67 mmol L^−1^ triacylglycerol, 3% (w/V) gum arabic, 2 mmol L^−1^ CaCl_2_, 2.5 mmol L^−1^ Tris–HCl and 150 mmol L^−1^ NaCl, dispersed in distilled water [87] and emulsified with a handheld mixer (400 watts, Royal Philips Electronics, Amsterdam, NH, Netherlands) at high speed, initially for 10 min and then for an additional 2 min immediately before use. The free fatty acids released during the reaction were titrated automatically to pH 7.5 in a Metrohm 718 STAT Titrino potentiometric titrator (Metrohm, Herisau, AR, Switzerland) with 0.05 mol L^−1^ NaOH, for 5 min. The reactions were carried out in a glass vessel at 30°C (unless otherwise stated) with 20 mL of substrate emulsion and 200 μL of solution buffer (2.5 mmol L^−1^ Tris–HCl pH 7.5, 2 mmol L^−1^ CaCl_2_) containing 0.025 mg of purified Lip-LifG9. All measurements were performed in triplicate and results are expressed as the average of these triplicate measurements ± the standard error of the mean. One unit (U) of lipase activity was defined as the release of 1 μmol of fatty acid per minute. The effects of pH (5.0 to 11.0, at 30°C) and temperature (20 to 60°C, at pH 7.5) on the activity of Lip-LifG9 were determined using tricaprylin as the substrate. The substrate emulsion and reaction conditions were the same as described above, except that the titration point was set at the stated value. Corrections were made for the partial dissociation of octanoic acid, assuming a pK_a_ of 4.89.

The activity of Lip-LifG9 was also determined against several triacylglycerols (triolein, tricaprylin and tributyrin and commercial oils from olive, corn, canola, palm, sunflower, castor, coconut and soybean). The emulsions were prepared with the corresponding triacylglycerols and oils, with the reactor maintained at pH 7.5 and 30°C.

The temperature stability of Lip-LifG9 was investigated by determining the residual activity, measured at 30°C and pH 7.5, after 1.5 h incubation at values from 0 to 80°C. To determine the pH stability, Lip-LifG9 was incubated for 1.5 h at 30°C in the buffers citrate (pH 5.5 and 6.0), MES (pH 5.5 to 7.0), citrate-phosphate (pH 6.5 and 7.0), Tris–HCl (pH 7.5 to 9.0) and glycine-NaOH (pH 9.0 to 10.0), at 50 mmol L^−1^ concentration, with the residual activity determined at pH 7.5 and 30°C. Tricaprylin was used as the substrate in both residual activity determinations.

The stability in hydrophilic organic solvents was determined by incubating Lip-LifG9 for 1.5 h at 30°C in aqueous solutions containing the solvents dimethylsulfoxide (DMSO, log P −1.3), acetonitrile (log P −0.33), ethanol (log P −0.24), acetone (log P −0.23), isopropanol (log P 0.28) and tetrahydrofuran (THF, log P 0.49) at the concentrations of 25, 50 and 75% (V/V). A solution of Lip-LifG9 (10 μL) containing 0.025 mg of protein was added to 2 mL of solvent solution. After the incubation, 40 μL of the medium was taken and added directly to the reaction vessel of the pH-Stat, where the residual activities against tricaprylin were determined at pH 7.5 and 30°C. 100% activity was taken as that obtained after incubation in an aqueous buffer (50 mmol L^−1^ Tris–HCl, pH 7.5) solution for 1.5 h.

### Nucleotide sequence accession number

The *lipG9* and *lifG9* nucleotide sequences reported here are available in the GenBank database under the accession numbers [GenBank:KM023399] and [GenBank:KM023400], respectively.

## Authors’ original submitted files for images

Below are the links to the authors’ original submitted files for images. Authors’ original file for figure 1 Authors’ original file for figure 2 Authors’ original file for figure 3 Authors’ original file for figure 4 Authors’ original file for figure 5 Authors’ original file for figure 6 Authors’ original file for figure 7 Authors’ original file for figure 8
